# **IUCrJ** launches electron crystallography section

**DOI:** 10.1107/S2052252522002251

**Published:** 2022-03-01

**Authors:** Peter R. Strickland

**Affiliations:** a Editorial Office, International Union of Crystallography, 5 Abbey Square, Chester, CH1 2HU, United Kingdom

**Keywords:** electron crystallography

## Abstract

The launch of an electron crystallography section in **IUCrJ** is announced.

Recent years have seen the rapid development and expansion of electron crystallography, much of it highlighted at the IUCr Congress in Prague last year. This has made it increasingly important to establish a home within IUCr journals for high-quality, high-impact papers in this field. The International Union of Crystallography is therefore pleased to announce the launch of a new section of **IUCrJ** dedicated to electron crystallography.

As announced in the January issue of the journal, Professor Xiaodong Zou (Stockholm University, Sweden) has been appointed as Main Editor of the electron crystallography section. Five new Co-editors will work with Professor Zou – Mauro Gemmi, Louisa Meshi, Peter Nellist, Jose Rodriguez and Junliang Sun – and will aim to attract articles reporting important electron crystallography results and breakthroughs to the journals. **IUCrJ** welcomes these new Co-editors and below we provide brief biographical details.

*Mauro Gemmi* (Istituto Italiano di Tecnologia, Pontedera, Italy) has worked in several electron microscopy labs in Europe (Stockholm University, Milan University, Institut Néel Grenoble) becoming an expert in the application of electron diffraction to structure solution problems. Since 2015 he has been the PI responsible for the Electron Crystallography research line of the Istituto Italiano di Tecnologia. He has been among the first to extensively use precession electron diffraction for solving crystal structures. His main research goal has been to apply electron diffraction to structural problems in any field of crystallography. His scientific dream is to see electron diffractometers in every crystallographic lab.

*Louisa Meshi* (Ben-Gurion University of the Negev, Beer-Sheva, Israel) specializes on structure solution of complex intermetallics applying precession electron diffraction (PED), convergent beam electron diffraction (CBED) and 3D electron diffraction tomography methods. In her laboratory, structure solution of these phases (appearing as nano-sized precipitates in metallic matrices) is combined with characterization of line and planar crystalline defects (using various electron imaging and diffraction methods), aiming at development of structural materials of tomorrow.

*Peter Nellist* FRS (University of Oxford, United Kingdom). His research centres on the applications and development of high-resolution electron microscope techniques, in particular scanning transmission electron microscopy (STEM) including 4D STEM diffraction-imaging techniques, *Z*-contrast imaging, electron energy-loss spectroscopy and applications of spherical aberration correctors. His technique development work includes ptychography, quantification of diffraction and image data, methods for the three-dimensional imaging and spectroscopy of materials using optical sectioning, and methods to allow high-resolution imaging and spectroscopy of radiation sensitive materials. Applications include Li-ion battery materials, hybrid organic–inorganic perovskites, polymer crystallinity and catalysts.

*Jose Rodriguez* (UCLA, Los Angeles, USA) studies the complex architecture of biological systems - from single biomolecules to cellular assemblies – at high resolution. His work is largely based on diffraction phenomena and combines computational, biochemical and biophysical experiments. The development of new methods is central to this work, particularly using emerging technologies in cryo-electron microscopy, nano and coherent X-ray diffraction, and macromolecular design. Combined, these tools can reveal undiscovered structures that broadly influence chemistry, biology and medicine.

*Junliang Sun* (Peking University, Beijing, China) works mainly on the development of structure characterization methods (electron crystallography and powder X-ray diffraction) and the synthesis of inorganic materials. Among the 245 approved zeolite topologies, he has been involved the discovery of ten topologies. In relation to structure characterization methods, Junliang and his co-workers have developed rotation electron diffraction which pushed the size limitation for single-crystal diffraction to tens of nanometres. Combined with powder X-ray diffraction, this has allowed series of difficult structure problems to be solved.

More information about the Editors, together with a selection of electron crystallography papers already published in the journal can be found at https://journals.iucr.org/m/services/editors_electron_cryst.html. We hope **IUCrJ** will become the natural home for your innovative research in electron crystallography and look forward to receiving your articles.

